# Albumin/Hyaluronic Acid Gel Nanoparticles Loaded with a Pyrimidine-Based Drug for Potent Anticancer Activity

**DOI:** 10.3390/gels11090759

**Published:** 2025-09-21

**Authors:** Sofia Teixeira, Débora Ferreira, Ligia R. Rodrigues, M. Alice Carvalho, Elisabete M. S. Castanheira

**Affiliations:** 1 Centre of Chemistry of University of Minho (CQUM), Campus de Gualtar, 4710-057 Braga, Portugal; 2 Physics Centre of Minho and Porto Universities (CF-UM-UP) and LaPMET (Laboratory of Physics for Materials and Emergent Technologies), University of Minho, Campus de Gualtar, 4710-057 Braga, Portugal; 3 Centre of Biological Engineering (CEB), University of Minho, Campus de Gualtar, 4710-057 Braga, Portugal; 4 LABBELS—Associated Laboratory, 4800 Guimarães, Portugal

**Keywords:** albumin-based nanoparticles, hyaluronic acid, pyrimidine-based drug, colorectal cancer, triple negative breast cancer

## Abstract

A pyrimidine-based compound (**PP**) was recently found to be a promising anticancer agent for colorectal and breast cancers. However, this compound exhibited low selectivity and poor water solubility. To address these challenges, albumin gel nanoparticles were used, where the gel matrix is formed by cross-linking of BSA molecules, allowing for a high concentration of this hydrophobic drug to be carried with no cytotoxicity to non-tumor cells. Functionalization with hyaluronic acid (HA) was employed to target CD44-overexpressing cancer cells, specifically triple-negative breast cancer (MDA-MB-231) and colorectal cancer cell lines (HCT 116). The gel nanoparticles present mean sizes below 250 nm, very low polydispersity, small aggregation tendency, and excellent colloidal stability in PBS buffer for a storage period of 30 days. Moreover, the drug-loaded particles showed high encapsulation efficiencies (above 85%) and sustained release profiles. Drug-loaded BSA/HA particles (**PP-HA-BSA-NPs**) revealed advantageous activity, presenting around 55% and 23% cell viability at a IC_50_ drug concentration for triple-negative breast cancer (the most aggressive breast cancer subtype) and colorectal cancer (second leading cause of cancer-related deaths), respectively. In conclusion, these nanoparticles outperform the ones without HA, demonstrating target capabilities, while retaining the drug’s anticancer activity and reducing the drug’s toxicity. These results are promising for future in vivo assays and clinical translational applications.

## 1. Introduction

Cancer remains a major challenge in modern society and is the second leading cause of death worldwide [1]. Anticancer drug effectiveness is often limited by issues such as poor solubility, stability, pharmacokinetics, toxicity, and low selectivity, leading to severe side effects [2,3,4]. While intravenous injection is the primary administration route, systemic circulation hinders tumor targeting and exacerbates side effects. Additionally, these drugs may undergo metabolism during transport, and low drug concentrations at the tumor periphery may prevent complete tumor eradication, potentially promoting metastasis [5].

To address these limitations, several drug delivery systems have been developed and implemented in cancer and inflammation therapies, including polymeric gels, inorganic nanoparticles, and proteins [6,7,8]. Compared to the administration of free drugs, encapsulation in a nanocarrier offers several advantages, including enhanced drug solubility and stability, improved tumor targeting due to the enhanced permeability and retention (EPR) effect, protection from degradation in the bloodstream, reduced side effects, and improved pharmacokinetic properties [4,5,9,10].

Albumin-based nanoformulations have been studied as targeted therapy systems for several diseases, with cancer being the primary focus of research [11]. These nanoparticles (NPs) can incorporate and transport both hydrophilic and poorly water-soluble drugs through aqueous medium, exhibiting a higher loading cargo than liposomes [12,13,14]. Despite BSA being more rigid than HSA, both proteins exhibit a similar size and environment of drug sites [15]. A wide range of antitumor drugs has been tested in BSA nanocarriers, including doxorubicin [16], paclitaxel [17], and 5-fluorouracil [18]. Moreover, these NPs offer various benefits, including preferential accumulation and uptake in tumors and inflamed tissue, as well as biocompatibility, high stability, biodegradability, non-immunogenicity, and non-toxicity [19,20]. To improve the specificity for tumors, nanoformulations have been functionalized to target overexpressed receptors in cancer cells [21].

Surface antigen differentiation group 44 (CD44) is a transmembrane, non-kinase, single-chain glycoprotein that has been extensively studied in recent years. Clinical and preclinical studies have demonstrated its role as a marker of progression and resistance to therapy in several types of cancer [22,23], particularly in breast [24] and colorectal [25] cancers. Consequently, this receptor serves as an active target for tumor-targeted therapy, using hyaluronic acid (HA), a natural polysaccharide with excellent biodegradability and biocompatibility, as a ligand [22,26,27,28]. Moreover, functionalization with HA enables hyaluronidase and pH-sensitive drug release [20].

**Figure 1:**
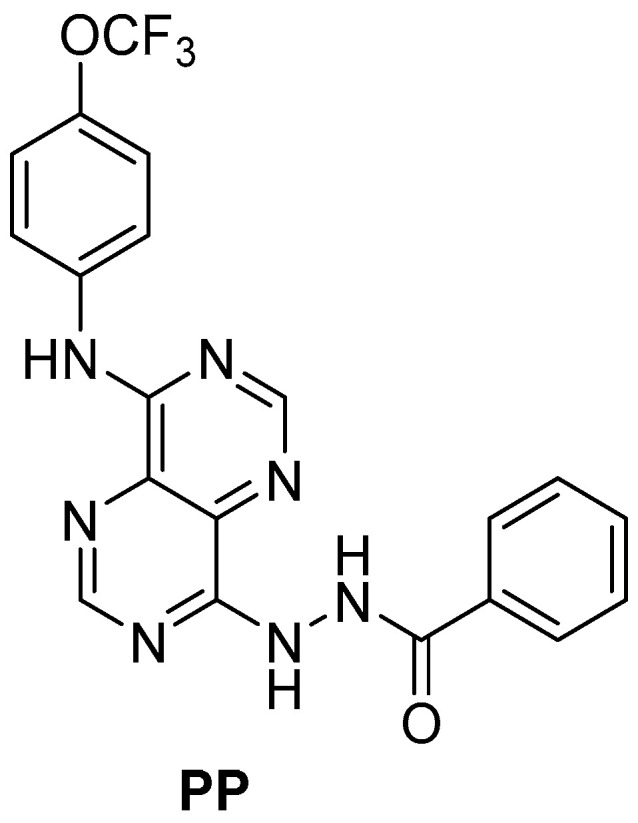
Structure of the new pyrimidine-based drug.

In a previous work, a novel pyrimidine-based drug (**PP**), was designed, and its in vitro anticancer activity was assessed. The compound **PP**, like many anticancer drugs, presents a heterocyclic planar core. This core has an aryl amine in C8 and an aryl hydrazide in C4 as substituents. The heterocyclic nucleus and the aryl units allow hydrophobic π-π intermolecular interactions, and the amine/hydrazide units permit hydrogen bond (hydrogen bond donor/acceptor) and polar interactions with biological targets fundamental for biological activity. The compound **PP** showed potent anticancer activity but poor water solubility (Figure 1) and was encapsulated in liposomal formulations. One of the obtained formulations showed comparable toxicity to **PP** against cancer cells and low toxicity [29]. However, no functionalization was used to specifically target cancer cells, and the **PP**-loaded formulations were obtained at a 16 µM **PP** concentration. Since albumin is a high abundant protein in human organisms and has a high affinity for hydrophobic drugs [30], in this work, **PP**-loaded albumin-based gel nanoformulations were developed to enhance drug loading. Functionalization with HA was performed for targeting CD44, which is overexpressed in colorectal (HCT 116) and triple-negative breast cancer (MDA-MB-231) cells. The drug-loaded formulations were characterized based on size and polydispersity, encapsulation efficiency, and release profiles. The anticancer activity was assessed in colorectal and triple-negative breast cancer cells, along with cytotoxicity in non-tumor BJ-5ta cells.

## 2. Results and Discussion

### 2.1. Drug-Loaded Albumin-Based Gel Nanoformulations Are Monodisperse and Exhibit Long Term Stability

The albumin-based gel nanoformulations were obtained by using the desolvation method [31] due to its low time-consuming reaction and reproducibility. The gelated matrix is formed by cross-linking of BSA molecules to increase the nanoparticulate system’s stability [14]. Among the two approaches for drug binding to albumin (covalent bonding or hydrophobic interaction), the **PP** antitumor compound passively binds to albumin through hydrophobic interactions within the albumin hydrophobic cavity [11]. HA was combined with drug-loaded albumin nanoparticles (NPs) for targeting modification, as ligands can bind to albumin through either covalent or non-covalent bonds. It has been described that the active amino group on the surface of albumin NPs may bind covalently via amide bonds with the carboxylic groups of HA [20].

Albumin-based gel nanoparticles (**BSA-NPs** and **HA-BSA-NPs**) and drug-loaded nanoparticles (**PP-BSA-NPs** and **PP-HA-BSA-NPs**), with varying concentrations of **PP**, were characterized based on their hydrodynamic size, polydispersity index (PDI), and zeta potential values, using Dynamic and Electrophoretic Light Scattering (Table 1). The hydrodynamic size values of non-loaded **BSA-NPs** and **HA-BSA-NPs** were 238 ± 2 nm and 192 ± 4 nm, respectively. When comparing the sizes of **PP-BSA-NPs** to their corresponding gel formulations without any drug, no significant impact was observed with an increasing drug concentration. Meanwhile, in **PP-HA-BSA-NPs**, a slight increase in size was detected. The diameter of both drug-loaded formulations remained below 250 nm, a size range favorable for tumor accumulation via the EPR effect [32]. The slight difference in the size of both nanoformulations is not expected to influence biodistribution and cellular uptake.

Moreover, the nanoformulations exhibit low polydispersity index (PDI) values ranging from 0.1 to 0.21, indicating monodisperse systems with minimal aggregation.

Albumin has an isoelectric point (pI) of 4.25 and carries a dense negative charge at physiological pH [20]. Both **PP-BSA-NP** and **PP-HA-BSA-NP** systems showed negative values of zeta potential in water and phosphate buffer, with a lower negative charge in the latter. Overall, **PP-BSA-NPs** and **PP-HA-BSA-NPs** did not show significant differences when compared to the placebo nanoformulations under the same conditions.

**Figure 2:**
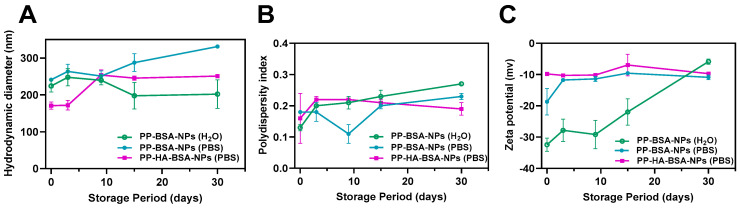
Stability assay of albumin-based formulations (**PP-BSA-NPs** and **PP-HA-BSA-NPs)** based on nanoparticles’ hydrodynamic size (**A**), size distribution (**B**), and zeta potential (**C**) over 30 days of storage at 4 °C. The stability of the formulations is compared to the original values (measured at day 0). Results are presented as the means and standard deviations (SDs) of the assays.

Among the several colloidal drug delivery systems used in cancer therapy, protein-based nanocarriers are recognized for their great storage stability. Particularly, the stability of albumin nanoparticles is attributed to the dense negative charges of albumin [20,33]. The stability of **PP-BSA-NPs** and **PP-HA-BSA-NPs** in phosphate buffer was monitored during storage at 4 °C for 30 days by DLS, measuring the variation in particles’ hydrodynamic size, size distribution, and zeta potential relative to the initial values (day zero) (Figure 2). The stability of **PP-BSA-NPs** was also investigated in pure water, with the nanoparticles maintaining a consistent size over time until the thirtieth day (Figure 2A). However, PDI exhibited a slight increase (Figure 2B), and the zeta potential decreased from −32.4 to −5.9 mV, indicating possible nanoparticles’ aggregation over time (Figure 2C). In PBS, **PP-BSA-NPs** showed only minor changes in size, PDI, and surface charge until day 15. A more prominent size increase was observed from day 9 to day 30. PDI values remained around 0.2, with small variations, while the surface charge remained stable until day 30.

**PP-HA-BSA-NPs** in PBS showed a slight increase in size between days 3 and 9, remaining stable thereafter. Their low polydispersity and consistently negative surface charge indicate minimal aggregation over time. Comparing both formulations, it can be seen that HA functionalization enhanced the stability of drug-loaded NPs.

Freeze-drying of nanoparticles can lead to aggregation and an increase in NP size. To prevent this, cryoprotectants such as sugars or polyols (glucose, maltose, sucrose, lactose, mannitol, and sorbitol) are used [34,35]. Trehalose and sucrose have shown the highest stabilization of albumin NPs after freeze-dying and subsequent reconstitution when compared to other cryoprotectants in several studies [34,36]. Cryoprotectants at a concentration of 2% *w*/*v* were tested for freeze-drying albumin NPs, with sucrose demonstrating the best performance [35]. After lyophilization, sucrose preserved the integrity and reduced aggregation of albumin nanoparticles, outperforming other cryoprotectants, because it has a lower glass transition temperature (T_g_ around −35 °C) than other excipients like mannitol (T_g_ ~−30 °C) and trehalose (T_g_ around −31 °C) [36], which helps prevent aggregation. Additionally, unlike glucose and lactose, sucrose is non-reducing, meaning it does not undergo Maillard reactions with amino groups in proteins, which minimize degradation or cross-linking during storage. As a result, there was less size increase and better redispersibility according to the results obtained [35,36].

Water removal from the albumin-based nanoformulations was necessary to increase concentration and facilitate handling for in vitro testing. Therefore, 2% (*w*/*v*) sucrose was applied to the albumin NP suspensions before lyophilization. Hydrodynamic size and PDI were evaluated after resuspension in water (Table 2), showing that all the nanoformulations exhibited minor variations in size. The PDI of **BSA-NPs** and **HA-BSA-NPs** remained unchanged, whereas **PP-BSA-NPs** and **PP-HA-BSA-NPs** showed a reduction in PDI from 0.2 to 0.1.

**Figure 3:**
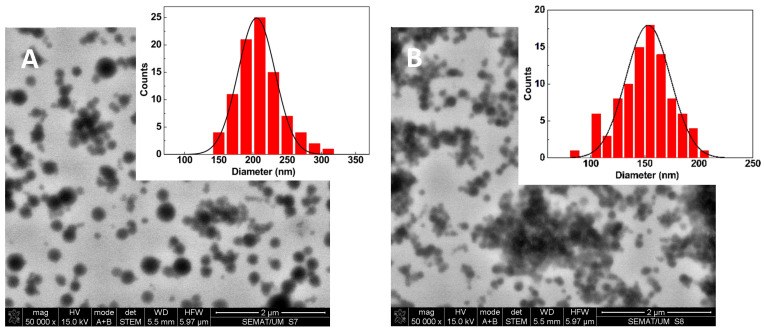
STEM images of lyophilized **PP-BSA-NP** (**A**) and **PP-HA-BSA-NP** (**B**) gel formulations and corresponding size histogram fitted to a Gaussian distribution.

STEM images were obtained for drug-loaded formulations containing 2% sucrose after freeze-drying and resuspension in water. **PP-BSA-NPs**, shown in Figure 3A, display spherical particles with sizes around 200 nm. The fitting of a Gaussian distribution to the size histogram, with a calculated mean diameter of 205 ± 27 nm, is in very good agreement with the results determined by DLS.

The reduction in the size of the drug-loaded BSA/HA nanoparticles with 2% sucrose (relative to the unloaded NPs and without sucrose) obtained by DLS was confirmed in STEM (Figure 3B). Here, the fitting to a Gaussian distribution to the size histogram allows for a mean diameter of 157 ± 21 nm to be obtained, which is slightly lower than the values of the hydrodynamic diameters obtained using the DLS technique (which was expected considering the drying process in SEM).

The gel nanoparticles’ stability in water may also be improved by the presence of sucrose, and this was assessed during storage at 4 °C for 30 days (Figure S1 in Supplementary Information). In the presence of sucrose, the NPs showed small variations in size, with a slight increase in PDI over time. Even so, the polydispersity remained below 0.3, indicating that the particles remained roughly monodisperse throughout the 30-day period. The zeta potential showed some fluctuations over time, but when compared to the results of stability previously observed for **PP-BSA-NPs**, the NPs did not suffer a significant decline in surface charge. The results suggest low aggregation of NPs in the presence of sucrose.

### 2.2. Albumin-Based NPs Allow High Encapsulation Efficiencies and Sustained Drug Release

The encapsulation efficiency, EE(%), of the antitumor compound in albumin-based nanoparticles were determined through fluorescence emission measurements using the calibration curve represented in Figure S2 (Supplementary Information). Compared to previous obtained liposomal formulations [29], albumin-based NPs allowed us to obtain higher amounts of the final compound. The resulting formulations achieved good to excellent encapsulation efficiencies, ranging from 84.6 to 98.5% (Table 3).

**Figure 4:**
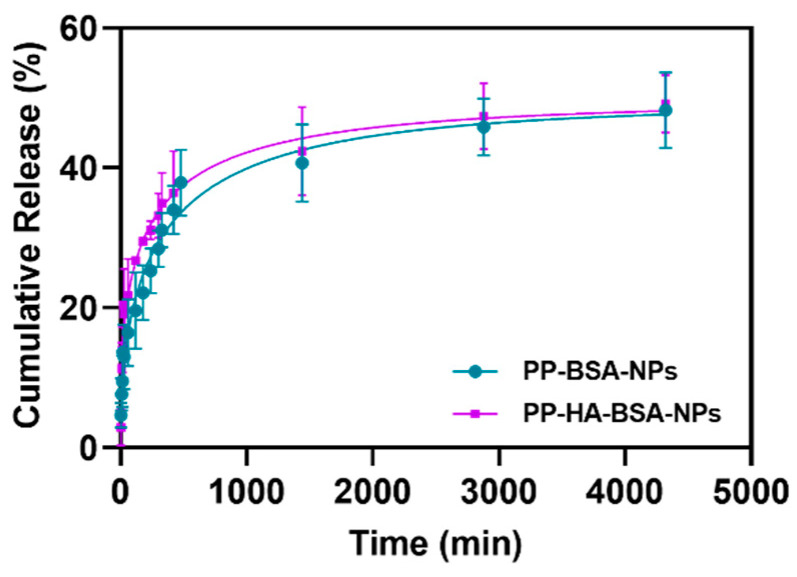
Cumulative release of antitumor compound from drug-loaded albumin-based gel nanoformulations (**PP-BSA-NPs** and **PP-HA-BSA-NPs**) fitted to the Weibull model. Results presented as mean and standard deviation of replicate assays.

The drug release profiles were assessed at room temperature over 72 h at pH = 7.4. The release kinetics of **PP** compound from albumin-based gel nanoformulations were fitted to the first-order kinetic model [37], to the Weibull model [38], and to the Korsmeyer–Peppas model [39] (Table S1 in Supplementary Information). Both **PP-BSA-NPs** and **PP-HA-BSA-NPs** exhibited similar drug release behavior over time (Figure 4), with approximately 50% of the drug being released to the aqueous medium within three days. The release profiles showed the best fit to the Weibull model (*R*^2^ = 0.928 for both nanosystems), following a Fickian diffusion with *b* values below 0.75 [38] (Table S1). The fittings of the experimental results to the first-order kinetic model and to the Korsmeyer–Peppas model are poor, exhibiting low coefficients of determination (Table S1).

The Korsmeyer–Peppas model is used to describe the release of a drug from a nanosystem (usually polymeric), being particularly useful when the release mechanism is unknown or complex, involving multiple phenomena like diffusion and polymer erosion [39]. Here, this model does not fit well the experimental data, pointing to a non-complex release mechanism dominated by Fickian diffusion, as inferred from the fit to the Weibull model.

Moreover, the drug release profiles were evaluated at physiological pH (pH = 7.4), which provides an initial understanding of sustained release under neutral conditions. It is expected that the release profile would be accelerated in the slightly acidic tumor microenvironment (pH~6.5) and particularly under lysosomal conditions (pH~5) due to enhanced hydrolysis of the albumin/HA network and enzymatic degradation by hyaluronidases and proteases [20,40].

### 2.3. PP-Loaded BSA/HA Gel Nanoparticles Exhibit Potent Anticancer Activity in Colorectal and Triple-Negative Breast Cancer Cells

The cytotoxicity of albumin-based nanoformulations was evaluated against two cancer cell lines, MDA-MB-231 and HCT 116, and a non-tumor cell line, BJ-5ta. As shown in Figure S3 (Supplementary Information), the cell viability in the presence of placebo (non-loaded) NPs remained above 70%, indicating no evidence of toxic effects on either cancer or normal cells, in accordance with ISO 10993-5 [41].

**Figure 5:**
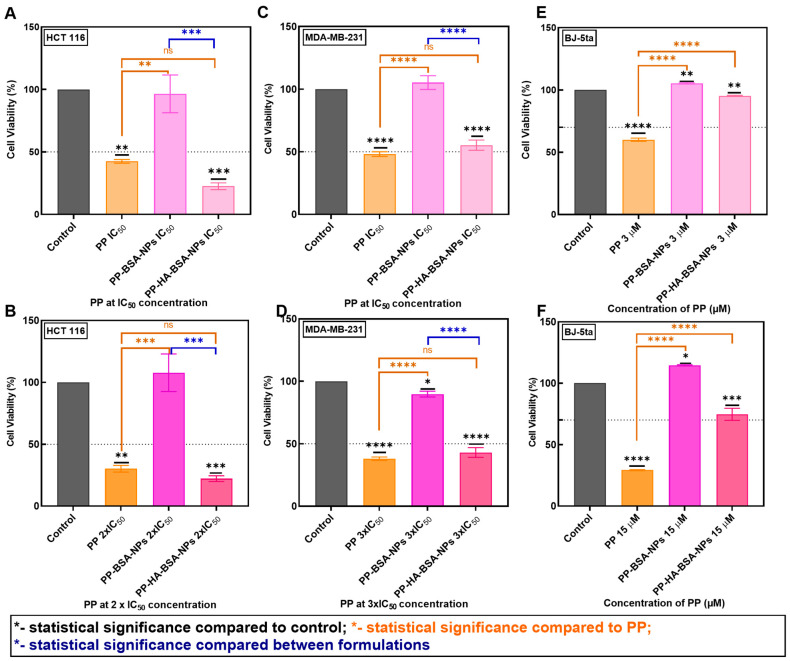
Assessment of the viability of HCT 116 (**A**,**B**), MDA-MB-231 (**C**,**D**), and BJ-5ta (**E**,**F**) after 48 h of exposure to drug-loaded albumin-based gel nanoformulations (**PP-BSA-NPs** and **PP-HA-BSA-NPs**). HCT 116 (**A**,**B**) exposure to **PP** and PP-loaded albumin-based gel nanoformulations with **PP** at IC_50_ and 2-fold IC_50_ concentration, respectively. MDA-MB-231 (**C**,**D**) exposure to **PP** and PP-loaded albumin-based nanoformulations with **PP** at IC_50_ and 3-fold IC_50_ concentration, respectively. BJ-5ta (**E**,**F**) exposure to **PP** and PP-loaded albumin-based gel nanoformulations at **PP** concentrations of 3 µM and 15 µM, respectively. Cell viability was determined using the MTT colorimetric assay. The cell viability of **PP** was normalized to DMSO, and the cell viability of PP-loaded albumin-based nanoformulations was normalized to untreated cells. One-way ANOVA indicates statistically significant differences within the group assessed using Tukey’s post hoc test. ns (non-statistical significance) *p* > 0.05, * *p* ≤ 0.05, ** *p* ≤ 0.01, *** *p* < 0.001, and **** *p* < 0.0001. Black * represents statistical significance of **PP** or PP-loaded formulations relative to control. Orange * represents statistical significance of PP-loaded formulations to the **PP** drug. Blue * represents statistical significance between PP-loaded formulations.

Previously, **PP** was identified as a promising anticancer drug, presenting inhibitory concentrations of 50% of cells (IC_50_) values of 2.04 ± 0.45 and 5.24 ± 0.24 µM on HCT 116 and MDA-MB-231 cell lines, respectively [29]. In this work, the cytotoxic effects of **PP** compound and **PP**-loaded albumin-based nanoformulations were evaluated at IC_50_, 2-fold IC_50_, or 3-fold IC_50_ in the same cancer cell lines (Figure 5). **PP-BSA-NPs** showed no considerable anticancer effect compared to free **PP** at IC_50_ (*p* ≤ 0.01) and 2-fold IC_50_ (*p* < 0.001) concentrations for HCT 116 cells (Figure 5A,B), and at IC_50_ (*p* < 0.0001) and 3-fold IC_50_ (*p* < 0.0001) for MDA-MB-231 cells (Figure 5C,D). In contrast, **PP-HA-BSA-NPs** demonstrated higher activity comparable to the free compound at both tested concentrations, with no statistically significant differences (*p* > 0.05) (Figure 5).

**Figure 6:**
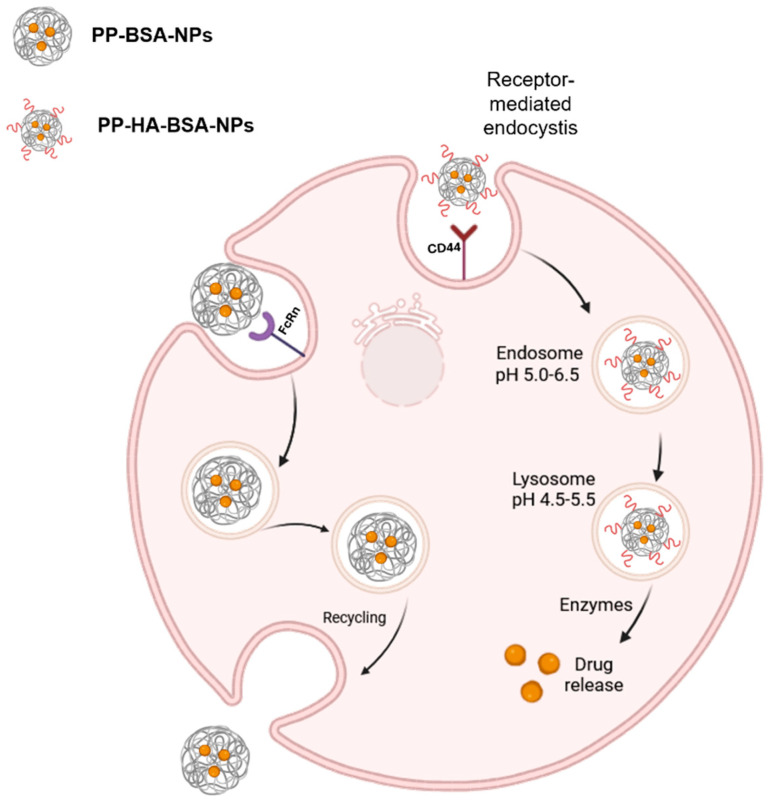
Schematic representation of the potential internalization and recycling of pyrimidopyrimidine-loaded bovine serum albumin nanoparticles (**PP-BSA-NPs**) via neonatal Fc receptor (FcRn), as well as internalization of pyrimidopyrimidine-loaded bovine serum albumin nanoparticles functionalized with hyaluronic acid (**PP-HA-BSA-NPs**) through CD44 receptors. The latter process involves lysosomal degradation of the NPs, followed by intracellular drug release. Figure created in BioRender [48].

The low anticancer activity of **PP-BSA-NPs** in vitro can be attributed to slow or inefficient cellular internalization, which relies on non-specific endocytosis mechanisms and sustained release processes. These factors may result in insufficient drug concentrations within the cells during the assay [33,42]. Moreover, when albumin is internalized via pinocytosis, it can be recognized and combined with neonatal Fc receptor (FcRn) receptors in low pH lysosomes, where it is recycled back to the extracellular space, avoiding degradation. Therefore, since the nanoparticles are mainly composed by albumin, they may also be recognized by FcRn receptors, escaping lysosomal degradation without releasing the drug (Figure 6) [11,13,34,43,44]. Moreover, the degradation process of albumin NPs can be hindered by a high degree of crosslinking and a large particle size [42]. Notably, MDA-MB-231 cells have been reported to overexpress FcRn, increasing the likelihood of **PP-BSA-NPs** being recognized by these receptors and escaping degradation, further contributing to their limited effectiveness [45].

On the other hand, CD44-overexpressed receptors on the cell surface can bind to the HA component of **PP-HA-BSA-NPs**, promoting active and efficient receptor-mediated endocytosis. This process facilitates HA/BSA nanocarriers’ uptake into endosomes. Within lysosomes, HA is degraded by hyaluronidases, which subsequently triggers the degradation of the entire NPs by lysosomal proteases, releasing the encapsulated drug (Figure 6) [46,47]. Hence, functionalizing NPs with HA enhanced their activity compared to non-functionalized ones. This improvement is attributed to the recognition of the particles by CD44-expressing cells, their degradation, and the resulting higher intracellular drug accumulation. It must be emphasized that CD44 is overexpressed in MDA-MB-231 and HCT 116 cancer cells at much higher levels than in healthy tissues, which provides a therapeutic window for selective targeting. Additionally, the enhanced permeability and retention (EPR) effect may further favor nanoparticle accumulation in tumor tissues rather than in normal organs.

The toxicity of albumin-based gel nanoformulations was analyzed using BJ-5ta cells (Figure 5E,F). Even at higher concentrations of the encapsulated compound in **PP-BSA-NPs** and **PP-HA-BSA-NPs**, cell viability remained above 70%, indicating a non-cytotoxic effect according to ISO 10993-5 [41] (Figure 5). These results suggest that **PP-HA-BSA-NPs** are excellent nanocarriers for the antitumor compound **PP** since it retains high anticancer activity comparable to the free drug, while reducing its toxicity towards normal BJ-5ta cells. This formulation demonstrates an excellent safety profile and aligns with reported findings, where drug-loaded albumin gel nanoformulations functionalized with HA have shown to retain or increase activity while improving the drug’s safety profile [20,21,40].

While our study highlights the strong in vitro performance of **PP-HA-BSA-NPs** in the studied cancer cells, we anticipate that these NPs may offer advantages in efflux pump-overexpressing cells. Nanoparticle-mediated delivery enables receptor-mediated endocytosis (via CD44 in MDA-MB-231 and HCT 116), bypassing some classical efflux mechanisms associated with multidrug resistance. Moreover, the sustained intracellular release of **PP** from lysosomal degradation of the nanocarriers may help maintain therapeutic concentrations within resistant cells.

Nevertheless, some challenges in future in vivo assays, such as stability in plasma, immune recognition, and pharmacokinetics, must be considered. Even so, albumin- and HA-based systems are generally known for their biocompatibility and favorable circulation profiles, which may help mitigate these issues. Future preclinical studies will be essential to confirm the safety, stability, and tumor-targeting potential of **PP-HA-BSA-NPs** in vivo.

## 3. Conclusions

In this work, albumin-based gel nanoformulations were prepared and characterized for the delivery of a novel antitumor pyrimidine-based compound (**PP**), which was recently synthesized. Both drug-loaded non-functionalized and HA-functionalized nanoparticles (**PP-BSA-NPs** and **PP-HA-BSA-NPs**) exhibited good physicochemical properties, including sizes below 250 nm, low polydispersity, and a negative surface charge, with a low tendency for aggregation. The albumin-based particles also showed good colloidal stability when stored at 4 °C, particularly in PBS and when functionalized with HA. The results indicated that **PP** did not affect these properties as no significant differences were observed compared to the placebo (unloaded) nanoformulations. Both formulations presented high encapsulation efficiencies (> 84%), which are comparable with previously reported values for hydrophobic drugs encapsulated in albumin nanoparticles via desolvation methods [49].

DLS measurements and STEM images of lyophilized **PP-BSA-NPs** and **PP-HA-BSA-NPs** (after re-suspension) confirmed that lyophilization of the nanoformulations (with 2% sucrose) effectively preserved nanoparticles’ integrity and stability upon reconstitution.

In previous work [29], liposomes were prepared as drug delivery systems for the novel agent **PP**, which showed to be promising as carriers for this compound, increasing its solubility and preserving **PP** anticancer activity, while minimizing its toxicity. Here, we developed albumin-based nanoparticles, aiming to increase selectivity and loading cargo. **PP-BSA-NPs** exhibited low anticancer activity, likely due to ineffective intracellular drug release. In contrast, the use of HA as a targeting ligand in the albumin nanoparticles, **PP-HA-BSA-NPs**, significantly improved drug delivery in CD44-expressing triple-negative breast cancer and colorectal cancer cells. In fact, **PP-HA-BSA-NPs** effectively targeted these cancer cell lines expressing CD44 while retaining the high anticancer activity of the free drug, allowed a minimum loading of 10-fold the IC_50_ value, and showed an exceptional safety profile.

Future work will focus on the **PP** mechanism of action through in vivo testing of **PP-HA-BSA-NPS** to evaluate biodistribution, therapeutic efficacy, and safety. Additional studies, such as those focusing on mechanism cellular uptake, drug release, and potential co-delivery with synergistic agents, will also be carried out. This formulation represents a strong candidate for advancing targeted therapies against resistant cancers, such as the resistant triple-negative breast cancer.

## 4. Materials and Methods

### 4.1. Preparation of Albumin-Based Gel Nanoformulations

Bovine serum albumin (BSA) (supplier item: A9418, from Sigma-Aldrich/Merck, Steinheim, Germany), *N*-(3-Dimethylaminopropyl)-*N*′-ethylcarbodiimide hydrochloride (EDC) (supplier item: 03449, from Sigma-Aldrich/Merck, Steinheim, Germany), and hyaluronic acid (HA) (supplier item: 924474, from Sigma-Aldrich, St. Louis, MO, USA) were used as received.

BSA gel nanoparticles (**BSA-NPs**) were synthesized through a desolvation method described elsewhere [31]. Absolute ethanol (8 mL) was added dropwise at a constant flow rate (1 mL/min) into BSA aqueous solution (62.5 mg/mL) under constant stirring (700 rpm). Next, the cross-linking agent EDC (4.5 mg) was added to the reaction mixture and left stirring for three hours. The resulting colloidal suspension was centrifuged at 9000 rpm for 30 min and redispersed in ultrapure water, followed by centrifugation under the same conditions to obtain a pellet of **BSA-NPs** gel nanoparticles. To prepare **PP**-loaded BSA gel nanoparticles (**PP-BSA-NPs**), an ethanolic solution containing **PP** was added to the BSA gel nanoparticle solution, yielding final **PP** concentrations of 16, 32, or 48 µM.

HA-BSA gel nanoparticles (**HA-BSA-NPs**) were also prepared using the desolvation method with minor adaptations to the procedure previously used by the research group [50]. Absolute ethanol (2 mL) was added dropwise at a constant flow rate (1 mL/min) into the BSA aqueous solution (62.5 mg/mL) under constant stirring (700 rpm). Next, EDC (1.5 mg) was added to the reaction mixture, and the mixture was stirred for one hour. Afterwards, water (16 mL) and 1 mL of HA aqueous solution (24 mg/mL) were added under stirring. Five minutes later, EDC (13.5 mg) was added, and stirring continued for 2.5 h. The resultant colloidal suspension was centrifuged at 9000 rpm for 30 min and redispersed in ultrapure water to wash the particles. The centrifugation process was repeated to obtain a white pellet of **HA-BSA-NPs** gel nanoparticles. To prepare **PP**-loaded HA-BSA gel nanoparticles (**PP-HA-BSA-NPs**), a **PP** ethanolic solution was added to obtain a final **PP** concentration of 16 or 48 µM in **PP**-loaded BSA.

For in vitro assays in cell lines, the gel nanoformulations were lyophilized in aqueous media with 2% (*w*/*v*) sucrose. The solutions were frozen at −20 °C overnight and then lyophilized in a LaboGene ScanVac CoolSafe Pro (LaboGene A/S, Allerød, Denmark) −110 °C at approximately 0.8 mbar.

### 4.2. Characterization of Albumin-Based Gel Nanoformulations

The average hydrodynamic size, polydispersity index, and zeta-potential of the prepared gel nanoparticles (*n* = 3 independent runs) were measured in a Dynamic Light Scattering (DLS) equipment Litesizer^TM^ 500 from Anton Paar (Anton Paar GmbH, Graz, Austria), with a 40 mW semiconductor laser diode (λ = 658 nm), a backscattering geometry (angle of 175°), and a controlled temperature of 25 °C. Scanning electron microscopy (SEM) images in transmission mode (STEM) were recorded using a NanoSEM—FEI Nova 200 (FEI company, Hillsboro, OR, USA) operating at 15 kV, coupled to an Electron Dispersive Spectroscopic analyzer (EDS) and Electron Backscatter Diffraction EDAX—Pegasus X4M at SEMAT/UM (Serviços de Caracterização de Materiais, Guimarães, Portugal). Processing of the STEM images was performed using ImageJ software (version 1.53t, National Institutes of Health, NIH, Bethesda, MD, USA).

### 4.3. Characterization of Drug-Loaded Albumin-Based Gel Nanoformulations

#### 4.3.1. Equipment

Fluorescence spectroscopy measurements were performed to determine the encapsulation efficiency and release profiles of the antitumor compound. Fluorescence spectra were recorded using a Fluorolog 3 (HORIBA Jobin Yvon IBH Ltd., Glasgow, UK) spectrofluorimeter equipped with Glan-Thompson polarizers and double monochromators in excitation and emission. The excitation of the **PP** compound was set at λ_exc_ = 400 nm, and the emission spectra were collected between 410 nm and 650 nm.

#### 4.3.2. Drug Encapsulation Efficiency and Release Assays

The encapsulation efficiency ($EE\left(\%\right)$) of **PP** in the nanoformulations was obtained through fluorescence emission measurements and determined using Equation (1). For each system, three independent measurements were carried out, and standard deviations (SD) were calculated.

(1) $$EE\left(\%\right)=\frac{\left(\mathrm{total}\;\mathrm{concentration}-\mathrm{concentration}\;\mathrm{of}\;\mathrm{non}\text{-}\mathrm{encapsulated}\;\mathrm{drug}\right)}{\mathrm{total}\;\mathrm{concentration}}\times 100$$

After the preparation of the gelated matrix, the supernatant was collected and subjected to centrifugation at 4500 rpm for 20 min in appropriate Amicon^®^ Ultra Centrifugal filters (10 kDa, Merck, Darmstadt, Germany). The water was evaporated at 80 °C and replaced with ethanol. Then, the fluorescence of the resulting solution was measured to determine the drug concentration.

The dialysis method, using dialysis membranes of 10 K MWCO, was used to perform the release assays of the antitumor compound from the nanocarriers while stirring in an orbital shaker at room temperature. The concentration of released drug was measured at different times over 72 h. The fluorescence intensity was measured to determine the concentration of the released drug. The release profiles were fitted to a first-order kinetic model and to the Weibull model. The first-order kinetic model [37] follows Equation (2),

(2) $$F\left(\%\right)=M_{0}\times (1-e^{-kt})$$

where *F* (%) is the percentage of the released compound, *M*_0_ represents the total amount of compound released, *k* represents the first-order rate constant, and *t* is the time.

The Weibull model [38] is a distribution function, which expresses the compound fraction accumulated, where *M_t_* and *M_∞_* are cumulative amounts of drug released at time *t* and infinite time, respectively, in the solution, following Equation (3),

(3) $$\frac{M_{t}\;}{M_{\infty }}=1-\exp\left[-{at}^{b}\right]$$

where *a* is a parameter defining the timescale of the process, and *b* denotes the curve type shape parameter. For *b* > 1, transport follows a complex release mechanism; *b* ≤ 0.75 indicates Fickian diffusion (in either fractal or Euclidian spaces), and 0.75 < *b* < 1 indicates a combined mechanism (Fickian diffusion and Case II transport) [38].

The Korsmeyer–Peppas model is described by Equation (4),

(4) $$\frac{M_{t}}{M_{\infty }}=K_{s}t^{n}$$

in which $\frac{M_{t}}{M_{\infty }}$ is the fraction of drug released at time *t*, and *K_s_* is the rate constant. For a spherical geometry, when *n* < 0.45, the release mechanism is diffusion-controlled (Fickian diffusion), 0.45 < *n* < 0.89 is an anomalous transport, and *n* ≥ 0.89 indicates that the release is mainly driven by swelling or relaxation of network chains (case-II transport) [39].

### 4.4. Anticancer Activity

#### 4.4.1. Cell Culture and Solutions

The colorectal cancer cell line HCT 116 (ATCC CCL-247, from American Type Culture Collection, Manassas, VA, USA) was cultured in Roswell Park Memorial Institute 1640 Medium (RPMI, Biochrom Ltd., Cambridge, UK), while the breast cancer cell line MDA-MB-231 (ATCC HTB-26, from American Type Culture Collection, Manassas, VA, USA) was grown in Dulbecco’s Modified Eagle Medium (DMEM, Biochrom Ltd., Cambridge, UK). Both media were supplemented with 10% (*v*/*v*) fetal bovine serum (FBS, Biochrom Ltd., Cambridge, UK) and 1% (*v*/*v*) penicillin-streptomycin (Biochrom Ltd., Cambridge, UK). The human fibroblast BJ-5ta cell line (ATCC CRL-2522, from American Type Culture Collection, Manassas, VA, USA) was grown in a 4:1 proportion of DMEM and Medium 199 (PAN-Biotech GmbH, Aidenbach, Germany), supplemented with 10% (*v*/*v*) FBS, 1% (*v*/*v*) penicillin-streptomycin, and 0.01% (*v*/*v*) hygromycin B (Sigma-Aldrich, St. Louis, MA, USA). The authenticity of all cell lines was confirmed, and these were also tested for mycoplasma. Cells were propagated in an incubator at 37 °C, 5% CO_2_, and 95% relative humidity. The cells were washed using phosphate-buffered saline (PBS) 1× at pH 7.4 and were detached using Trypsin/EDTA solution (Biochrom Ltd., Cambridge, UK). Cell counts were performed in a hemocytometer.

For 3-(4,5-Dimethylthiazol-2-yl)-2,5-Diphenyltetrazolium Bromide (MTT, Sigma Aldrich, St. Louis, MA, USA) experiments, logarithmically growing HCT 116 and MDA-MB-231 cell lines were seeded in 96-well plates at a concentration of 0.5 × 10^5^ cells/mL, and BJ-5ta cells were seeded at a concentration of 0.8 × 10^5^ cells/mL.

#### 4.4.2. Cytotoxicity Assays

To study the cytotoxic effect of placebo (non-loaded) albumin-based gel nanoformulations (**BSA-NPs** and **HA-BSA-NPs**), the cells were exposed at different concentrations of NPs and dissolved in 100 µL of the respective culture media at 37 °C for 48 h (a standard timeframe for evaluating anticancer activity in vitro). HCT 116 cells were exposed to **BSA-NPs** at 0.3 and 0.6 mg/mL and to **HA-BSA-NPs** at 0.37 and 0.74 mg/mL; MDA-MB-231 cells were exposed to **BSA-NPs** at 0.5 and 1.5 mg/mL and to **HA-BSA-NPs** at 0.6 and 1.8 mg/mL; BJ-5ta cells were treated with **BSA-NPs** at 0.3 and 1.5 mg/mL and **HA-BSA-NPs** at 0.37 and 1.8 mg/mL.

To evaluate the effect of **PP**-loaded albumin-based nanoformulations (**PP-BSA-NPs** and **PP-HA-BSA-NPs**) on HCT 116, MDA-MB-231, and BJ-5ta cells, they were exposed to treatment with the **PP** compound at different concentrations, along with 1% (*v*/*v*) DMSO (control, obtained from Thermo Fisher Scientific, Waltham, MA, USA), and dissolved in 100 µL of the respective culture media at 37 °C for 48 h. HCT-116 cells were treated with the **PP** compound at previously calculated IC_50_ [29] and 2-fold IC_50_ concentrations (2 and 4 µM, respectively). MDA-MB-231 cells were exposed to the **PP** compound at previously calculated IC_50_ [29] and 3-fold IC_50_ concentrations (5 and 15 µM, respectively). BJ-5ta cells were exposed to treatment with the **PP** compound at 3 and 15 µM concentrations.

After the incubation period, the culture media were discarded from each well, followed by the addition of MTT solution (100 µL, 0.5 mg/mL). After incubation of the plates for 2 h, the supernatant was removed, and DMSO (100 µL) was added to solubilize the purple formazan crystals for 15 min at room temperature. Cell viability was determined based on the absorbance measured at 570 nm in a microplate reader (Cytation 3, BioTek Instruments, Winooski, VT, USA). The absorbance of the untreated cells or DMSO-treated cells was considered 100% cell viability. Triplicate assays were carried out, and all conditions were performed in at least triplicate wells.

### 4.5. Statistical Analysis

At least three independent experiments were conducted for each assay. Results are shown as mean ± standard mean of error (SEM). GraphPad Prism 8 (GraphPad Software, Inc., La Jolla, CA, USA) was used to produce a graphical representation of the results, and statistical significance was analyzed by one-way analysis of variance (ANOVA) to compare control vs. experimental groups or to compare experimental groups. Statistical significance was established for all comparisons at the following significance levels: ns (non-statistical significance, *p* > 0.05); * *p* ≤ 0.05; ** *p* ≤ 0.01; *** *p* < 0.001; and **** *p* < 0.0001.

## Figures and Tables

**Table 1:** Hydrodynamic size, polydispersity (PDI), and zeta potential of placebo (non-loaded) albumin gel nanoformulations (**BSA-NPs** and **HA-BSA-NPs**) and drug-loaded nanoformulations (**PP-BSA-NPs** and **PP-HA-BSA-NPs**).

Albumin Nanoformulations	Medium	Concentration of PP (µM)	Hydrodynamic Diameter ± SD ^1^ (nm)	PDI ± SD	Zeta Potential ± SD ^1^ (mV)
**BSA-NPs**	Water	-	238 ± 2	0.21 ± 0.03	−35.9 ± 0.9
**PP-BSA-NPs**	Water	16	224 ± 17	0.13 ± 0.01	−32.4 ± 2
**PP-BSA-NPs**	Phosphate buffer	16	242 ± 3	0.21 ± 0.04	−15.8 ± 5
**PP-BSA-NPs**	Water	32	208 ± 1	0.07 ± 0.02	−28.4 ± 2
**PP-BSA-NPs**	Water	48	214 ± 10	0.20 ± 0.06	−24.5 ± 5
**HA-BSA-NPs**	Water	-	192 ± 4	0.21 ± 0.02	−26.6 ± 1
**PP-HA-BSA-NPs**	Phosphate buffer	16	205 ± 49	0.18 ± 0.07	−9.0 ± 1
**PP-HA-BSA-NPs**	Water	48	245 ± 1	0.21 ± 0.02	−26.1 ± 0.5

^1^ SD: standard deviation of three replicate assays.

**Table 2:** Hydrodynamic size and polydispersity (PDI) of placebo albumin gel nanoformulations (**BSA-NPs** and **HA-BSA-NPs**) and drug-loaded nanoformulations (**PP-BSA-NPs** or **PP-HA-BSA-NPs**) with (after lyophilization) or without (before lyophilization) 2% (*w*/*v*) sucrose.

Albumin Nanoformulations	Hydrodynamic Diameter ± SD ^1^ (nm)	PDI ± SD ^1^
**BSA-NPs**	238 ± 2	0.21 ± 0.03
**BSA-NPs** + 2% sucrose (*w*/*v*)	266 ± 23	0.22 ± 0.02
**PP-BSA-NPs**	198 ± 3	0.15 ± 0.02
**PP-BSA-NPs** + 2% sucrose (*w*/*v*)	198 ± 1	0.10 ± 0.01
**HA-BSA-NPs**	192 ± 4	0.21 ± 0.02
**HA-BSA-NPs** + 2% sucrose (*w*/*v*)	225 ± 2	0.22 ± 0.03
**PP-HA-BSA-NPs**	245 ± 8	0.20 ± 0.03
**PP-HA-BSA-NPs** + 2% sucrose (*w*/*v*)	185 ± 1	0.12 ± 0.01

^1^ SD: standard deviation.

**Table 3:** Drug encapsulation efficiency in albumin-based gel nanoformulations.

Nanoformulations	Final Concentration of Compound (µM)	EE(%) ± SD
**PP-BSA-NPs**	16	94.8 ± 4
**PP-BSA-NPs**	32	96.1 ± 1
**PP-BSA-NPs**	48	98.2 ± 0.7
**PP-HA-BSA-NPs**	16	84.6 ± 6
**PP-HA-BSA-NPs**	48	98.5 ± 0.3

## Data Availability

The data presented in this study is contained within the article and Supplementary Materials. Further inquiries can be directed to the corresponding authors.

## Supplementary Materials

The following supporting information can be downloaded at https://www.mdpi.com/article/10.3390/gels11090759/s1, 1. Results: 1.1. Stability of albumin-based gel nanoformulations with 2% (*w*/*v*) of sucrose; 1.2. **PP** calibration curve of fluorescence intensity vs. concentration; 1.3. Fitting of drug release profiles; 1.4. Biological assays. Figure S1: Assay of stability of albumin-based gel formulations **PP-BSA-NPs** and **PP-HA-BSA-NPs** in the presence of sucrose: hydrodynamic size (A), polydispersity (B), and zeta potential (C) for 30 days upon storage at 4 °C; Figure S2: Calibration curve of fluorescence intensity of **PP** vs. concentration; Figure S3: Assessment of the viability of HCT 116 (A), MDA-MB-231 (B), and BJ-5ta (C) after 48 h of exposure to placebo albumin-based gel nanoformulations. Table S1: Parameters obtained by fitting the release profiles of drug-loaded albumin-based nanoformulations to the first-order kinetic model, Weibull model, and Korsmeyer–Peppas model, with the respective coefficients of determination (*R*^2^).

## Abbreviations

The following abbreviations are used in this manuscript:

PP	Pyrimido[5,4-*d*]pyrimidine compound
NPs	Nanoparticles
HA	Hyaluronic acid
BSA	Bovine serum albumin
EDC	*N*-(3-Dimethylaminopropyl)-*N*’-ethylcarbodiimide hydrochloride
PBS	Phosphate-buffered saline
DLS	Dynamic light scattering
PDI	Polydispersity
EE	Encapsulation efficiency
STEM	Scanning electron microscopy in transmission mode
