# Supplementary material for: Albumin/Hyaluronic Acid Gel Nanoparticles Loaded with a Pyrimidine-Based Drug for Potent Anticancer Activity
Source: Gels. 2025 Sep 21;11(9):759. doi: 10.3390/gels11090759 (PMC12469947; doi:10.3390/gels11090759)
Supplement: Supplementary file 1 [file gels-11-00759-s001.zip › gels-3848026-supplementary.pdf]

Electronic Supplementary Information (ESI)

Albumin/hyaluronic acid gel nanoparticles loaded with a pyrimidine-based drug for a potent anticancer activity

Sofia Teixeira [1,2], Débora Ferreira [3,4], Ligia R. Rodrigues [3,4], Maria Alice Carvalho [1,*] and Elisabete M. S. Castanheira [2,*]

1. Results

1.1. Stability of albumin-based gel nanoformulations with 2% (w/v) of sucrose

Figure S1 shows the variation of nanoparticles size, size distribution, and zeta potential for 30 days upon storage at 4 °C.

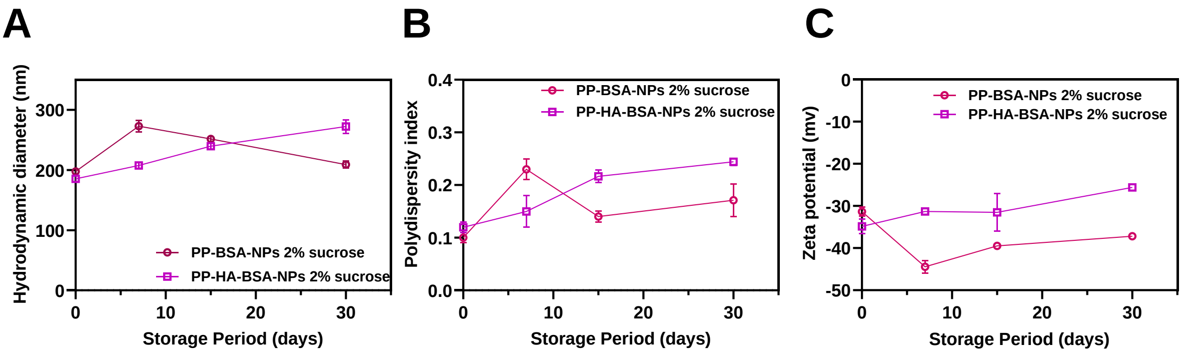

Figure S1. Assay of stability of albumin-based gel formulations **PP-BSA-NPs** and **PP-HA-BSA-NPs** in the presence of sucrose: hydrodynamic size (**A**), polydispersity (**B**), and zeta potential (**C**) for 30 days upon storage at 4°C. The stability of the formulations is compared to the original values (measured at day 0). Results presented as mean and standard deviation (SD) of three independent measurements.

1.2. PP Calibration curve of fluorescence intensity vs. concentration

Figure S2 shows the calibration curve of the **PP** compound fluorescence intensity vs. concentration.

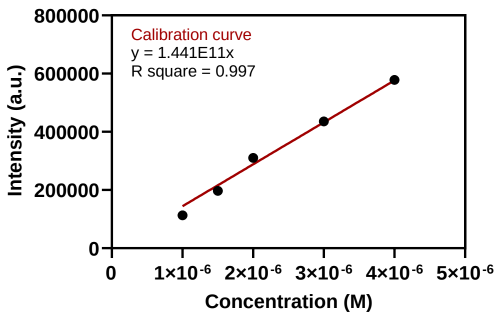

Figure S2. Calibration curve of fluorescence intensity of **PP** vs. concentration.

1.3. Fitting of drug release profiles

The results of the fitting of release profiles to first-order kinetics, Weibull and Korsmeyer-Peppas models are displayed in **Table S1**.

Table S1. Parameters obtained by fitting the release profiles of drug-loaded albumin-based gel nanoformulations to the first-order kinetic model, Weibull model, and Korsmeyer-Peppas model, with the respective coefficients of determination (R^2).

Albumin-based gel nanoformulations	First-order kinetics		Weibull			Korsmeyer-Peppas		
	k (min^{-1})	R^2	b	a	R^2	K_s	n	R^2
**PP-BSA-NPs**	0.00421	0.843	0.5078	0.05022	0.928	0.1046	0.2541	0.883
**PP-HA-BSA-NPs**	0.01468	0.758	0.4303	0.09783	0.928	0.1546	0.2086	0.878

1.4. Biological assays

Figure S3 shows the viability of HCT 116, MDA-MB-231 and BJ-5ta cells after exposure to **BSA-NPs** and **HA-BSA-NPs** over a 48-hour treatment period.

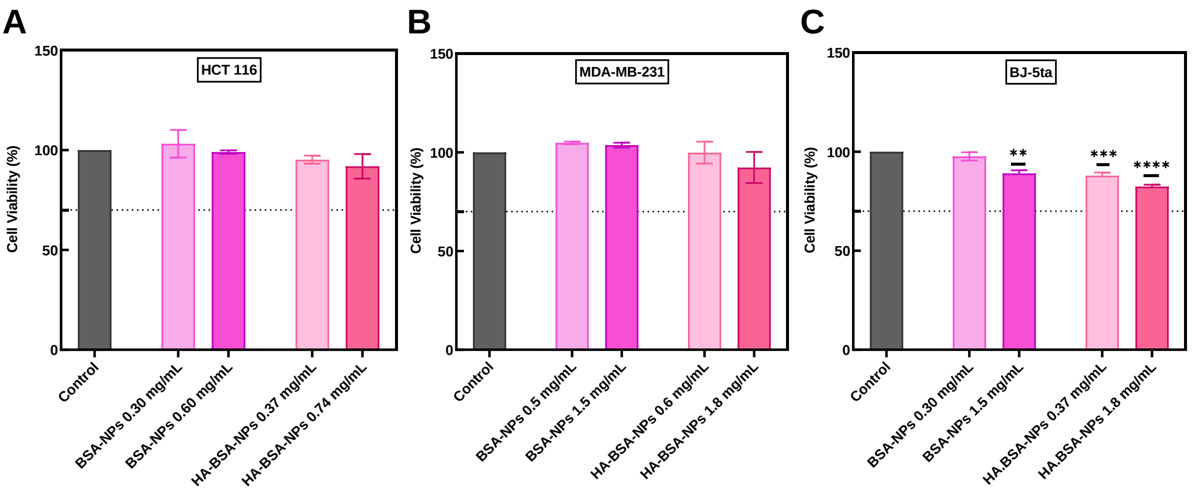

Figure S3. Assessment of the viability of HCT 116 (**A**), MDA-MB-231 (**B**) and BJ-5ta (**C**) after 48 hours exposure to placebo albumin-based gel nanoformulations. HCT 116 (**A**) exposure to **BSA-NPs** concentrations of 0.3 mg/mL and 0.6 mg/mL and to **HA-BSA-NPs** concentrations of 0.37 mg/mL and 0.74 mg/mL. MDA-MB-231 (**B**) exposure to **BSA-NPs** concentrations of 0.5 mg/mL and 1.5 mg/mL and to **HA-BSA-NPs** concentrations of 0.6 mg/mL and 1.8 mg/mL. BJ-5ta (**C**) exposure to exposure to **BSA-NPs** concentrations of 0.3 mg/mL and 1.5 mg/mL and to **HA-BSA-NPs** concentrations of 0.37 mg/mL and 1.8 mg/mL. Cell viability was determined using the MTT colorimetric assay and normalized to untreated cells. One-way ANOVA indicates statistically significant differences to control assessed by Dunnett's post-test, and denoted as follows: ns (non-statistical significance) $p > 0.05$, * $p \leq 0.05$, ** $p \leq 0.01$, *** $p < 0.001$, and **** $p < 0.0001$. No statistically significant differences were found for **BSA-NPs** and **HA-BSA-NPs** in HCT 116 and MD-MB-231 cells.
